# Supplementary material for: Farnesoid X Receptor Overexpression Decreases the Migration, Invasion and Angiogenesis of Human Bladder Cancers via AMPK Activation and Cholesterol Biosynthesis Inhibition
Source: Cancers (Basel). 2022 Sep 9;14(18):4398. doi: 10.3390/cancers14184398 (PMC9497084; doi:10.3390/cancers14184398)
Supplement: Supplementary file 1 [file cancers-14-04398-s001.zip › cancers-1803840-supplementary.pdf]

Supplementary Figures

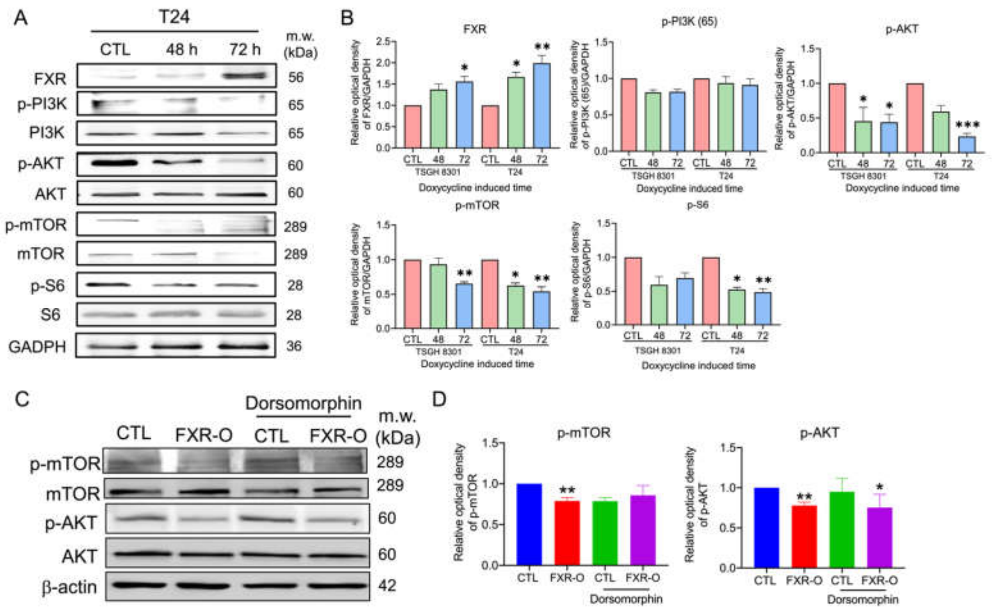

Supplementary Figure S1. The effect of FXR overexpression on the PI3K/AKT/mTOR pathway. (A) The levels of phosphatidylinositol-3-kinase (PI3K), AKT and mammalian target of rapamycin (mTOR) were analyzed by Western blotting in T24 cells after FXR overexpression for 48 and 72 h. GAPDH was used as the loading control. (B) The bar graphs show the relative quantitative analysis of these proteins. (C) The protein expression of p-mTOR, mTOR, p-AKT, and AKT in FXR-O T24 cells treated with or without dorsomorphin was analyzed by Western blot. β-actin was applied as the loading control. (D) The bar graphs show the relative quantitative analysis of p-mTOR and p-AKT. * $p < 0.05$; ** $p < 0.01$; *** $p < 0.001$ compared to the control group.

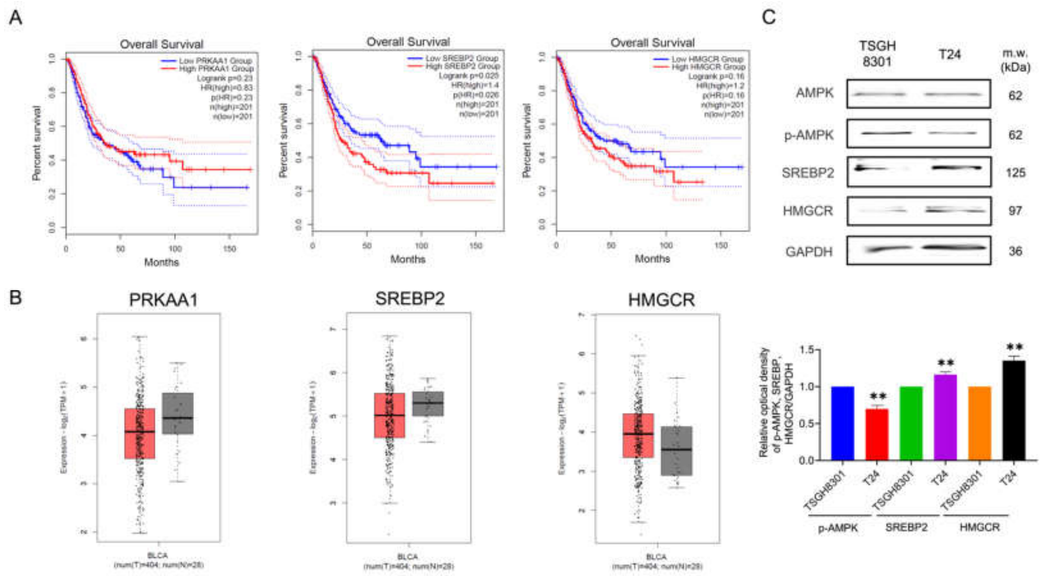

Supplementary Figure S2. The effects of PRKAA1, SREBP2 and HMGCR expression on overall survival in human bladder cancer patients and protein expression in bladder cancer cell lines. (A) Overall survival rate of bladder cancer patients with differential expression of the PRKAA1 (AMPK), SREBP2 and HMGCR genes in the TCGA database. (B) Scatter plots of the differential expression of the PRKAA1, SREBP2 and HMGCR genes in bladder cancer tissues (red plot) and adjacent normal tissues (gray plot) from the TCGA database. (C) The expression levels of p-AMPK, AMPK, SREBP2 and HMGCR in the bladder cancer cell lines were analyzed by Western blotting. GAPDH was used as a loading control. ** $p < 0.01$ compared with the TSGH8301 group.

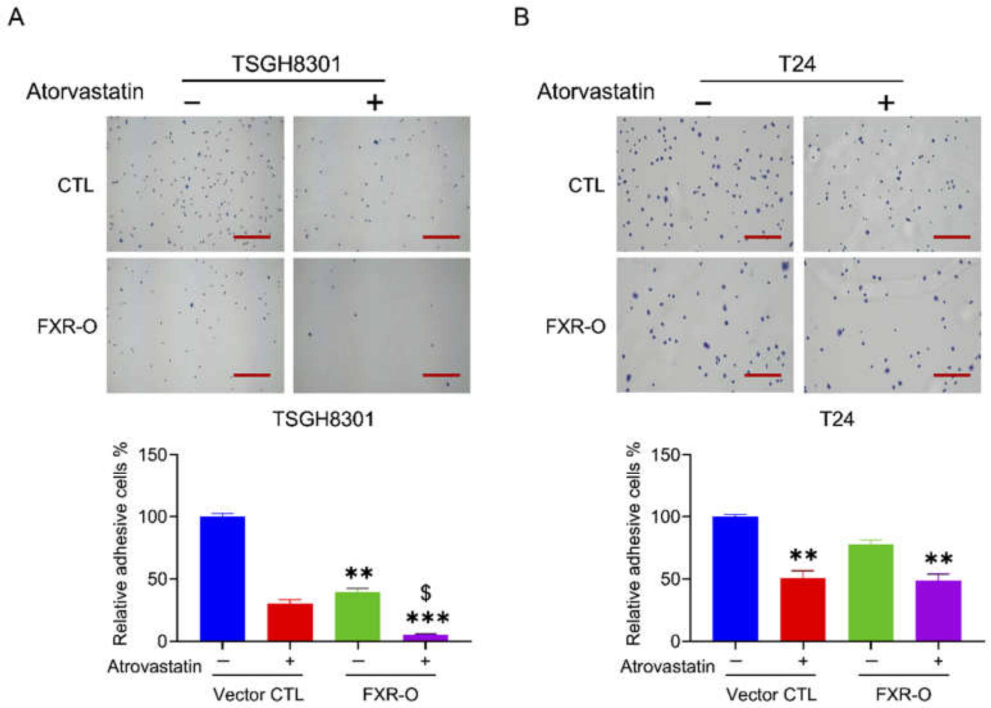

Supplementary Figure S3. The effect of FXR overexpression combined with atorvastatin treatment on adhesion. (A) Adhesion assays were performed in control and FXR-O TSGH8301 cells treated with or without atorvastatin. After 50 min of incubation, the adherent cells were stained and imaged. The lower panel shows the quantitative results. (B) Adhesion assays were performed in control and FXR-O T24 cells treated with or without atorvastatin. The lower panel shows the quantitative results. ** $p < 0.01$; *** $p < 0.001$ compared with the control group. ## $p < 0.01$, ### $p < 0.001$ compared to the control + atorvastatin group. $$ $p < 0.01$; $$$ $p < 0.001$ compared to the FXR-O group. Scale bar = 200 µm.

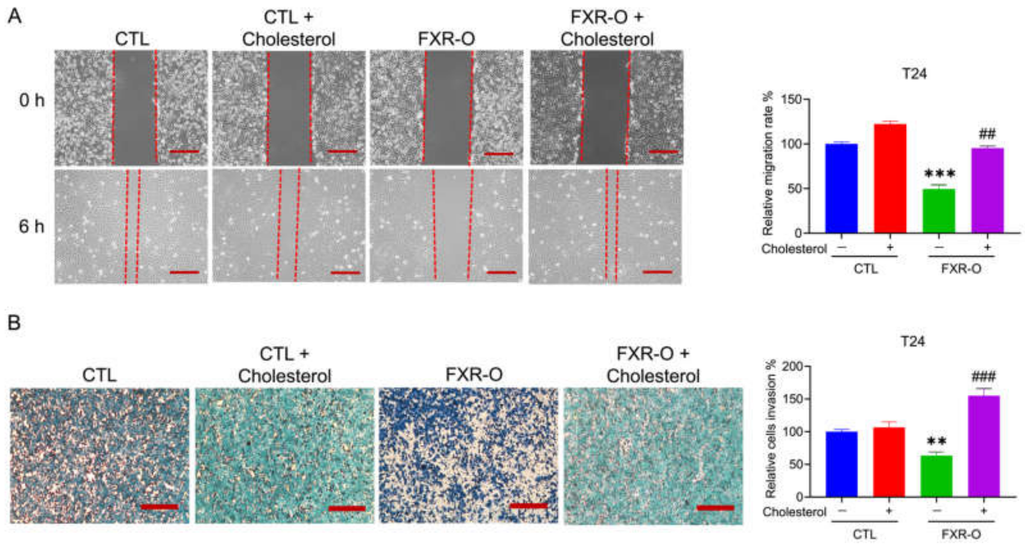

Supplementary Figure S4. The effect of cholesterol application on migration and invasion. (A) Wound healing migration and (B) Transwell invasion assay were performed after the addition of cholesterol (400 pg/mL) to the FXR overexpression group. ** $p < 0.01$; *** $p < 0.001$ compared with the control group. ## $p < 0.01$; ### $p < 0.001$ compared with the FXR overexpression group. Scale bar = 200 μm.

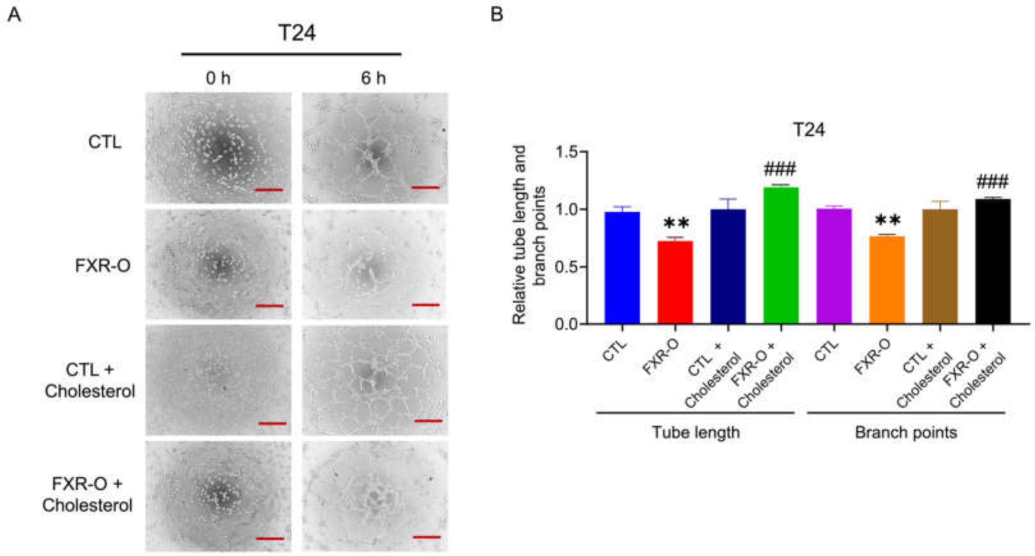

Supplementary Figure S5. Cholesterol application reversed the FXR overexpression-mediated inhibition of tube formation. (A) A tube formation assay was performed after the addition of cholesterol (400 pg/mL) to the FXR overexpression group. The total length of HUVECs was imaged and measured after 6 h of incubation. (B) The bar graphs show the branch point numbers and tube lengths. * $p < 0.05$; ** $p < 0.01$ compared with the control group. ### $p < 0.001$ compared with the FXR overexpression group. Scale bar = 200 μm.

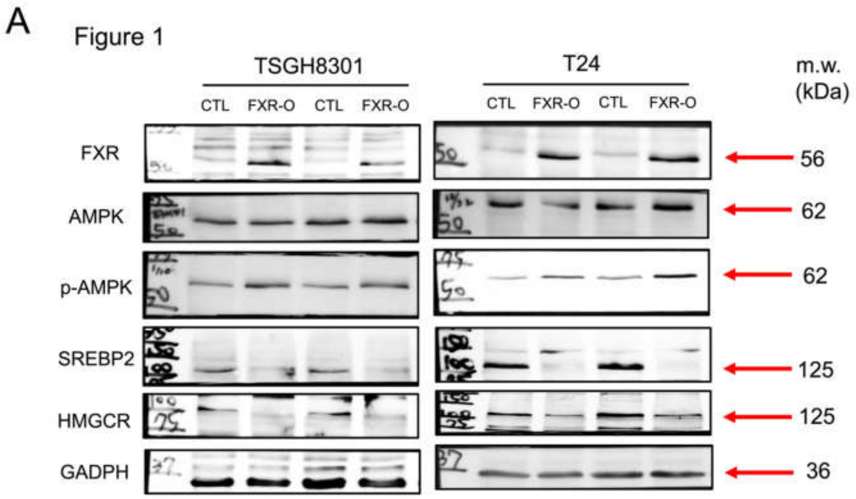

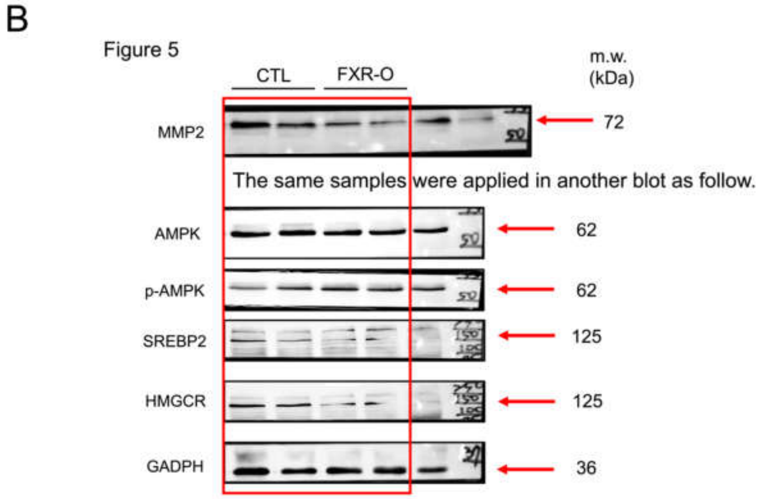

Supplementary Figure S6. Original blots. (A) Original blots for Figure 1 were shown. (B) Original blots for Figure 5 were shown.

Supplementary Table S1. The information of antibodies.

Name	Species	Brand	Cat NO.
GAPDH	Rb	CST	5174
FXR	Ms	SANTA CRUZ	sc-25309
p-AMPK	Rb	CST	2535
AMPK	Rb	CST	5831
SREBP2	Ms	SANTA CRUZ	sc-13552
HMGCR	Ms	SANTA CRUZ	sc-271595
MMP2	Rb	CST	13132
p-PI3K	Rb	CST	4228S
PI3K	Rb	CST	4292
p-AKT (T308)	Rb	CST	13038
AKT	Rb	Epitomics	1085-1
p-mTOR	Rb	CST	5536S
mTOR	Rb	CST	2972S
p-S6	Rb	CST	4858S
S6	Rb	CST	2217S
